# Comparison of quality of life and cosmetic result between open and transaxillary endoscopic thyroid lobectomy for papillary thyroid microcarcinoma survivors: A single‐center prospective cohort study

**DOI:** 10.1002/cam4.4766

**Published:** 2022-04-25

**Authors:** Tingting Li, Zhicheng Zhang, Weisheng Chen, Shitong Yu, Baihui Sun, Xiangqian Deng, Junna Ge, Zhigang Wei, Shangtong Lei, Guoxin Li

**Affiliations:** ^1^ Department of General Surgery & Guangdong Provincial Key Laboratory of Precision Medicine for Gastrointestinal Tumor, Nanfang Hospital, The First School of Clinical Medicine Southern Medical University Guangzhou Guangdong China

**Keywords:** quality of life, scar, thyroid cancer, transaxillary

## Abstract

**Background:**

Transaxillary endoscopic thyroidectomy has been introduced to achieve better cosmetic outcomes. However, the benefits of this technology on the patients’ health‐related quality of life (HRQoL) remain unclear. We aimed to investigate whether transaxillary endoscopic lobectomy is comparable to conventional open lobectomy in terms of QOL and cosmetic results in order to provide more evidence for establishing appropriate clinical decisions.

**Methods:**

Between August 2019 and May 2020, transaxillary endoscopic lobectomy and conventional open lobectomy were performed in 73 and 99 patients with papillary thyroid microcarcinoma, respectively. HRQoL was assessed at 1, 3, 6, and 12 months after surgery using the Thyroid Cancer‐Specific Quality of Life Questionnaire. The cosmetic outcomes were assessed 12 months after surgery using the Patient and Observer Scar Assessment Scale (POSAS).

**Results:**

No significant difference was observed in the surgical results between the two groups. However, the data showed that the average operative time and postoperative hospital stay of the transaxillary group were longer than those of the open group (*p* < 0.001). Both groups showed similar changes in the QOL scores over time. However, the transaxillary group had fewer complaints of the throat or oral problems at 1 month postoperatively than the open group (*p* < 0.001). During the follow‐up, the cosmetic results of scars in the transaxillary group were significantly better than those in the open group (*p* < 0.05). Patients who underwent transaxillary endoscopic lobectomy had higher overall satisfaction with their scar appearance, determined using POSAS, at 12 months postoperatively.

**Conclusions:**

The current findings suggest that transaxillary endoscopic lobectomy may offer better cosmetic and HRQoL outcomes.

## Graphical abstract

Transaxillary surgery has obvious advantages in terms of HRQoL and cosmetic effects. The role of transaxillary surgery as an alternative strategy for patients with PTMC is worthy of recognition.

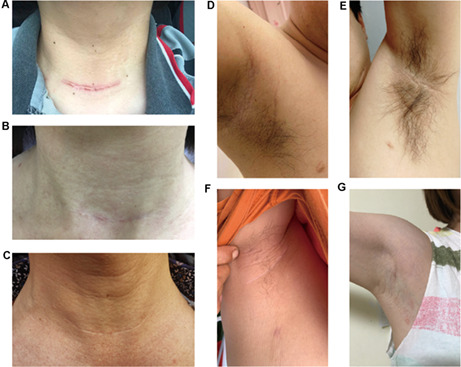

## 1 INTRODUCTION

The incidence of thyroid cancer has been continuously increasing in China and worldwide over the last few decades.^1^ From 2005 to 2015, the incidence of thyroid cancer substantially increased in China. The crude incidence rates of thyroid cancer were 13.17/105 in 2015 and 4.3/105 in 2005.^2^ With the increase in the popularity of ultrasound detection and the improvement of ultrasound detection technology, an increasing number of thyroid cancer patients have been clinically diagnosed, especially those with papillary thyroid microcarcinoma (PTMC) with a maximum diameter of ≤1 cm,^3^, ^4^ which is characterized by high morbidity, low mortality, and a high 10‐year survival rate.^5^, ^6^ Due to the high survival rate and longevity after treatment, the treatment focused on improving the quality of life of survivors. The known factors that can affect the quality of life of thyroid cancer survivors include voice and swallowing functions, postoperative pain, shoulder discomfort, sensory abnormalities, dyskinesia, and cosmetic effects.^7^, ^8^ An increasing number of young and female patients with PTMC often complain of ugly scars in the visible anterior neck area after conventional open lobectomy.^9^

Thus, increasing attention has been paid to investigating the new extra‐cervical “remote” approaches that can avoid scarring in the neck to improve the patients’ quality of life (QoL).^10^ One of the most common remote approaches is transaxillary endoscopic lobectomy.^11^, ^12^, ^13^ Using this technology, the development of neck scars can be avoided, thereby obtaining better cosmetic results.^10^, ^14^ In recent years, increasing evidence has shown that the complication rates of transaxillary endoscopic lobectomy are similar to those of open thyroid surgery, with comparable tumor control results and better cosmetic satisfaction rates.^15^, ^16^, ^17^, ^18^ A previous study had investigated the health‐related QoL (HRQoL) results of transaxillary endoscopic lobectomy in a small cohort and short postoperative follow‐up time.^8^ At the same time, some surgeons believe that transaxillary endoscopic lobectomy has some disadvantages compared with conventional open surgery. It is more invasive and results in slower postoperative recovery and poorer postoperative QoL.^19^, ^20^ Therefore, the HRQoL of patients undergoing transaxillary endoscopic lobectomy has not been fully studied.

In order to provide more evidence for establishing appropriate clinical decisions, we analyzed the postoperative HRQoL and cosmetic results of patients who underwent transaxillary endoscopic lobectomy and conventional open lobectomy using two different and verified questionnaires with longer postoperative follow‐up times.

## 2 MATERIALS AND METHODS

The data of consecutive patients with papillary thyroid microcarcinoma (PTMC) who underwent surgery were prospectively evaluated. The patients were grouped according to the type of surgery. After counseling on the surgical options, eligible patients and their families chose between transaxillary endoscopic lobectomy (transaxillary group) and conventional open surgery (open group). All patients were recruited from a single center. After screening based on the inclusive and exclusive criteria, all included patients were followed up for 1 year, and relevant data were collected for statistical analysis, including clinical and operative indicators, responses to the questionnaire (THYCA‐QOL), and scale (POSAS) scores, in order to compare the HRQoL results and cosmetic outcomes between the two different surgical procedures.

### 2.1 Patient selection

**FIGURE 1:**
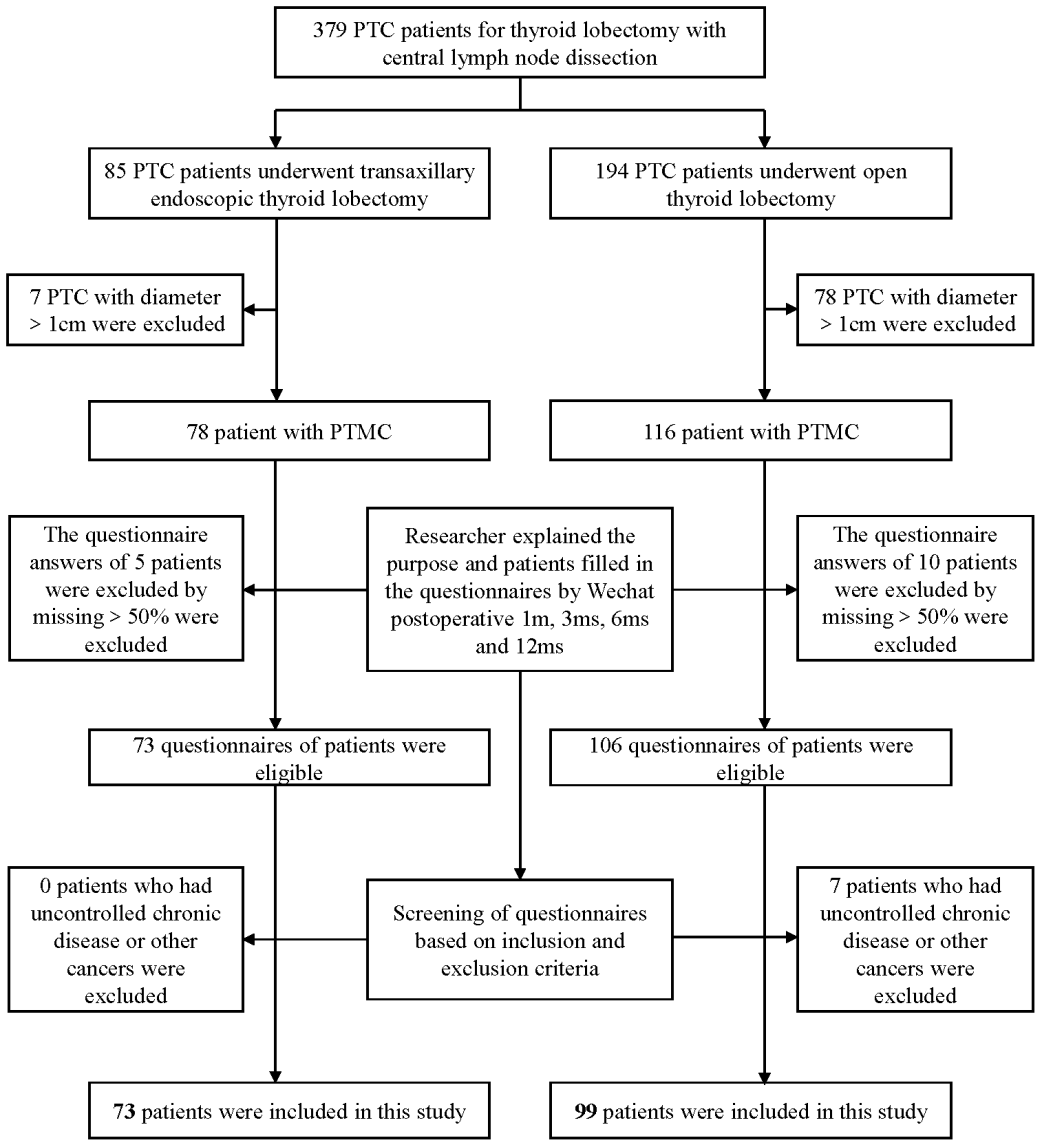
The flow chart of inclusion and exclusion and follow‐up process of the transaxillary endoscopic lobectomy and conventional open lobectomy group. PTC, papillary thyroid carcinoma; PTMC, papillary thyroid microcarcinoma

A total of 172 patients underwent unilateral thyroid lobectomy and central lymph node dissection using the gasless transaxillary or traditional open approach at Nanfang Hospital, the affiliated hospital of Southern Medical University, from August 2019 to May 2020 were enrolled in this study. The flowchart is presented in Figure 1. Patients with low‐risk differentiated microcarcinoma (isolated suspicious thyroid nodule with a maximum diameter of ≤1 cm detected by preoperative ultrasound), aged ≥18 years, who did not previously undergo cervical radiotherapy, with no neck lymph node metastasis, with no extra‐thyroid metastasis, with no distant metastasis, with noninvasive variants, and who had cosmetic needs requiring endoscopic surgery were included in the transaxillary group. Meanwhile, patients diagnosed with low‐risk differentiated microcarcinoma who had no cosmetic needs and refused to undergo endoscopic surgery were included in the open group. Patients with the following conditions were excluded: those (1) who previously underwent neck surgery or radiotherapy; (2) with other pathological conditions in the neck or shoulder; (3) with suspected tumors invading the adjacent organs, such as the recurrent laryngeal nerve (RLN), esophagus, trachea, or internal jugular vein; (4) with distant metastases; and (5) with uncontrolled chronic disease or other types of cancer.

Our study was approved by the institutional review committee of our hospital. Each participant provided informed consent before completing the questionnaires to assess the patients’ HRQoL and cosmetic outcomes.

### 2.2 Surgical procedure

For the gasless transaxillary endoscopic approach, the patient was placed in the supine position after the administration of general anesthesia and tracheal intubation. In order to slightly extend the neck, two thin sponge rolls were placed under the neck and shoulders. The affected arm was abducted at 90° to expose the axilla. A 3 cm long incision was created behind the anterior axillary fold. This incision was used to place a retractor in order to maintain the working space during surgery. A 0.5 cm incision was made 5 cm from the first incision where the instrument port of the endoscope was inserted. A 30° endoscope was placed in a 3 cm incision. Electrocautery was used to dissect the anterior neck between the pectoralis major muscle and subcutaneous soft tissue of the anterior chest. Once in the neck, the sternocleidomastoid muscle (SCM) and band muscles were identified. The space between the chest and clavicular bones of the SCM was determined. Afterward, the space was separated in order to expose the omohyoid muscle that slanted outward and downward. The deep cervical fascia was cut at the lateral edge of the subhyoid muscles to locate and enter the space between the sternal thyroid muscle and the thyroid. The retractor was lifted and hung to maintain the working space and expose the thyroid. The SCM was lifted, and the thyroid gland was pressed down. Under a 30°endoscope, an ultrasonic knife and grasping forceps were used to separate the surface of the thyroid gland from the surrounding soft tissues. An ultrasonic knife was used to identify and carefully cut the blood vessels near the upper pole of the thyroid. The superior parathyroid glands near the upper pole were identified and preserved. An ultrasonic knife was used to identify and cut the middle thyroid veins. The RLN was separated from the blood vessels under the thyroid. An ultrasound knife was used to cut the inferior thyroid arteries and veins close to the thyroid gland. The RLN was carefully dissected along its path until the cricothyroid junction was reached. The inferior parathyroid glands near the lower pole were identified and retained. The thyroid and central lymph nodes in front of the trachea were cut. Bleeding was stopped, and a drainage tube was inserted through the port incision. Finally, all surgical wounds were carefully closed layer by layer.

For the conventional open approach, a 5–10 cm lateral neckline incision was made at two finger widths above the sternum notch. The subplatysmal flap was lifted to the thyroid cartilage and down to the sternal notch. The band muscles were separated at the midline to expose the thyroid gland. The upper thyroid blood vessels were identified and ligated. The thyroid lobe was dissected while preserving the RLN. The middle and inferior thyroid vessels were cut. The thyroid lobe, isthmus, and central lymph nodes were also removed. A drainage tube was then inserted next to the incision. The surgical wound was carefully closed layer by layer.

### 2.3 Operative evaluation

Data on the critical indicators of the operation were collected and analyzed, including operative time, blood loss, tumor size, positive central lymph node, postoperative hospital stay, and complications, as they may affect the postoperative evaluation of patients.

### 2.4 Postoperative follow‐up

Follow‐up was performed at 1, 3, 6, and 12 months after surgery. The results of the questionnaires and scales were obtained using the WeChat App. The doctors sent the questionnaires and scales to each patient and explained the content of both tools through WeChat; the patients returned them after providing their responses.

**FIGURE 2:**
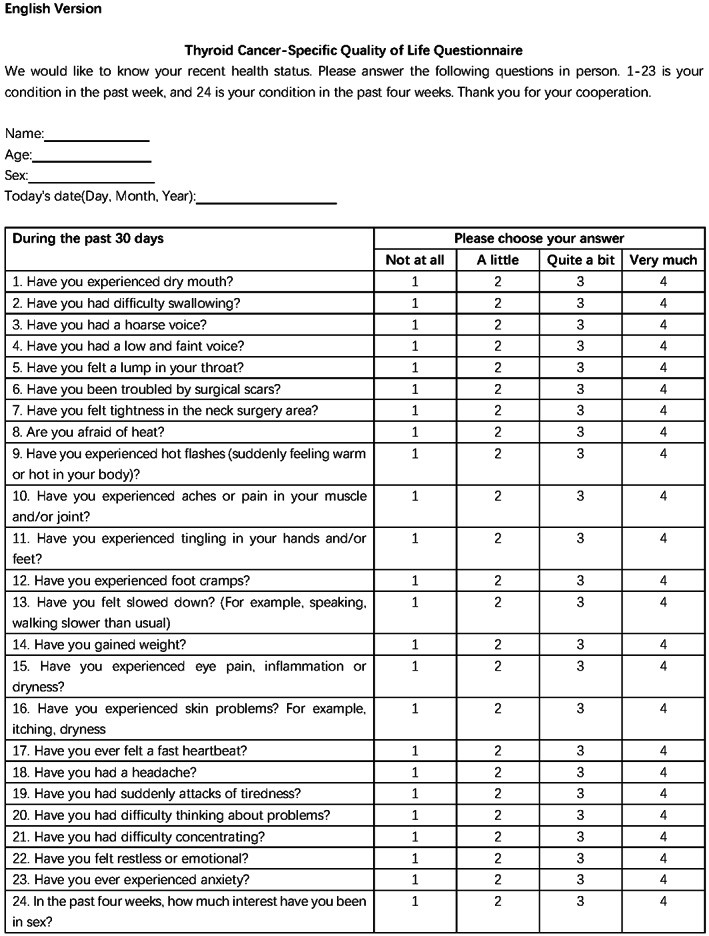
The English version of the Thyroid Cancer‐Specific Quality of Life Questionnaire (THYCA‐QOL)

The Chinese version of the THYCA‐QOL (Figure 2) was used to assess for thyroid‐specific symptoms caused by thyroid cancer or its treatment.^21^ It includes 24 questions that measure seven symptom domains (neuromuscular, voice, attention, sympathetic, throat/mouth, psychological, and sensory symptoms) and six single scales (scar, feeling cold, tingling sensation, weight gain, headache, and reduced sexual interest).^22^ The questionnaire had a strict timescale. Sexual interest items were answered based on each patient’s experience within the period of 4 weeks, while those other items were based on their experience within the period of within 1 week. All items were divided into four levels (1 = “not at all,” 2 = “a little,” 3 = “quite a bit,” and 4 = “very much”). All items were scored from 1 to 4 points. Higher scores indicated more complaints caused by the symptoms and worse QoL.

**FIGURE 3:**
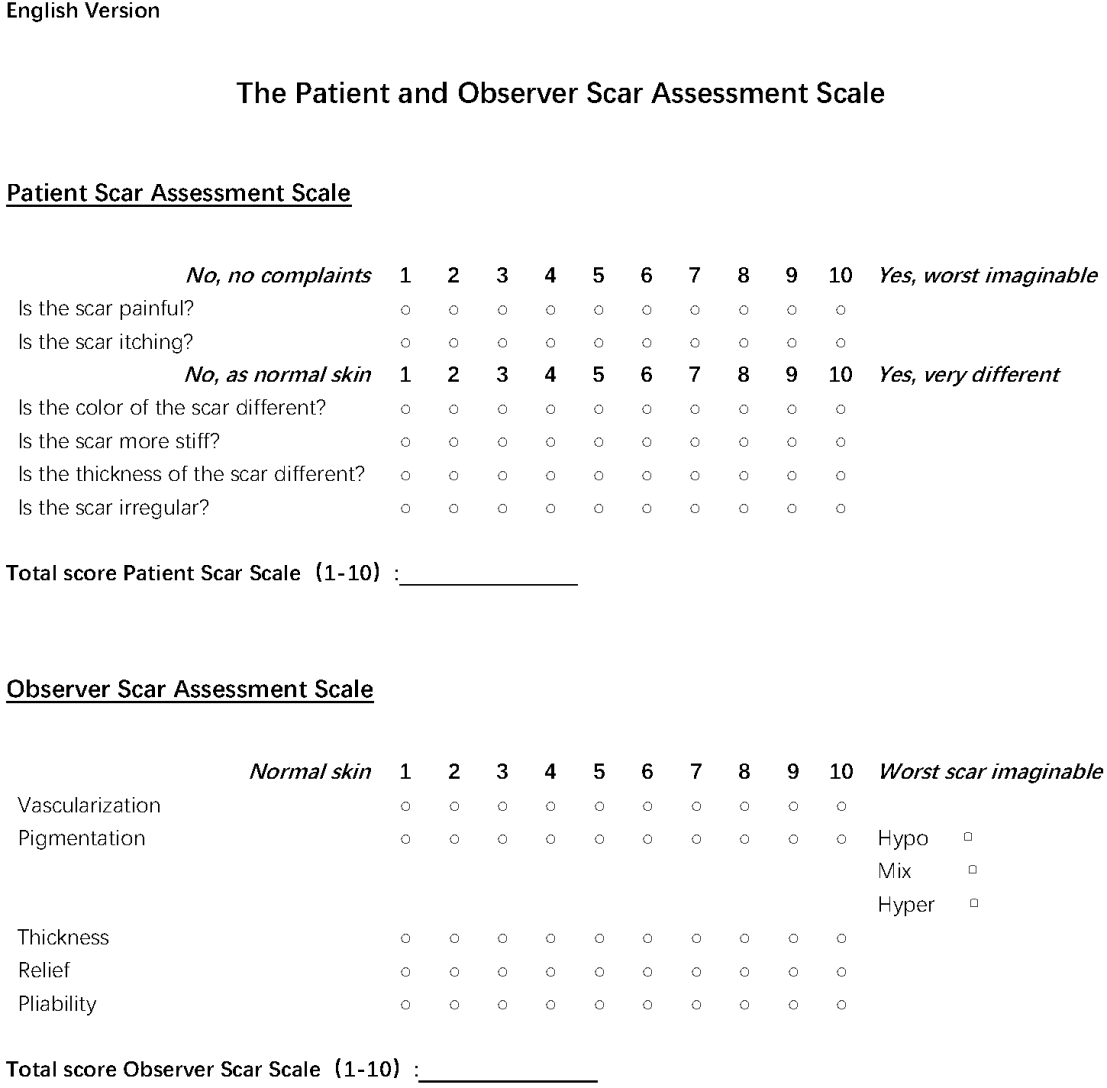
The English version of the Patient and Observer Scar Assessment Scale (POSAS)

The Patient and Observer Scar Assessment Scale (Figure 3) was used to assess for scarring 12 months after surgery. It consists of two scales: the patient scale contains six items, whereas the observer scale contains five items.^23^ All items on the two scales were scored numerically. Patients scored the characteristics of scar color, flexibility, thickness, relief, itching, and pain, whereas observers scored the scar vascularization, pigmentation, flexibility, thickness, and relief. In addition, according to the documents used in the Vancouver scale, pigmentation was described as hypopigmentation, hyperpigmentation, or mixed pigmentation (Figure 3). Observers were required to rate the blood vessel formation and pigmentation. Meanwhile, the patients were only required to rate the color of scars as they were expected to have difficulties in distinguishing vascularization from pigmentation. All items were rated from 1 to 10, of which 10 points reflected the worst scarring or sensation.

### 2.5 Statistical analysis

Categorical variables were expressed as numbers, while continuous variables were expressed as means and standard deviations. *T* test was used to compare continuous variables. All *p* values were two‐sided, and a *p* value of <0.05 was considered significant. Data and figures were analyzed and generated using SPSS software (version 24.0; IBM, Inc.) and GraphPad Prism version 7.0 (Graph Pad Software, Inc.).

## 3 RESULTS

### 3.1 Baseline characteristics of the patients and surgery

A total of 172 patients underwent unilateral thyroidectomy with central lymph node dissection, of whom 57 were men and 115 were women, with an average age of 37.94 ± 8.05 years (range: 21–57 years). Ninety‐nine patients chose the traditional open approach, whereas 73 patients chose the transaxillary endoscopic approach. No differences were observed in sex, age, or body mass index between the transaxillary and open groups. In terms of surgical indicators, no significant differences were observed in blood loss volume, tumor size, positive central lymph nodes, and complications between the two groups (Table 1). However, the average operation time and postoperative hospital stay in the transaxillary endoscopy group were significantly extended (113.01 ± 14.92 min vs.74.71 ± 16.55 min, *p* < 0.001; 3.41 ± 0.76 days vs. 2.86 ± 0.73 days, *p* < 0.001; Table 1). Of the 73 patients who underwent transaxillary surgery, two experienced hoarseness after surgery. However, they recovered within 4 weeks after the operation. In our study, none of the patients in the transaxillary group developed complications such as surgical site infection or chyle leakage.

**TABLE 1:** Baseline characteristics of papillary thyroid microcarcinoma patients in transaxillary group and open group

Characteristics	Transaxillary group (*n* = 73)	Open group (*n* = 99)	*p* value
Sex: *n* (%)			
Female	54 (74.0%)	61 (61.6%)	0.089
Male	19 (26.0%)	38 (38.4%)	
Age (mean ± SD; year)	36.63 ± 7.48	38.91 ± 8.36	0.066
BMI (mean ± SD; kg/m^2^)	21.38 ± 2.37	22.18 ± 2.84	0.052
Operative time (mean ± SD; min)	113.01 ± 14.92	74.71 ± 16.55	<0.001
Blood loss (mean ± SD; ml)	5.73 ± 2.69	6.34 ± 3.00	0.166
Tumor size (mean ± SD; mm)	0.58 ± 0.215	0.58 ± 0.24	0.889
Positive central lymph node (mean ± SD)	1.03 ± 2	1.22 ± 2.16	0.547
Postoperative hospital stay (mean ± SD; day)	3.41 ± 0.76	2.86 ± 0.73	<0.001
Complication: *n* (%)			
Bleed	2 (2.7%)	2 (2%)	0.757
Transient vocal cord paralysis	2 (2.7%)	5 (5.1%)	0.448
Surgical site infection	0	1 (1.0%)	0.389
Chyle leakage	0	1 (1.0%)	0.389

### 3.2 Statistical results of THYCA‐QoLquestionnaires

**FIGURE 4:**
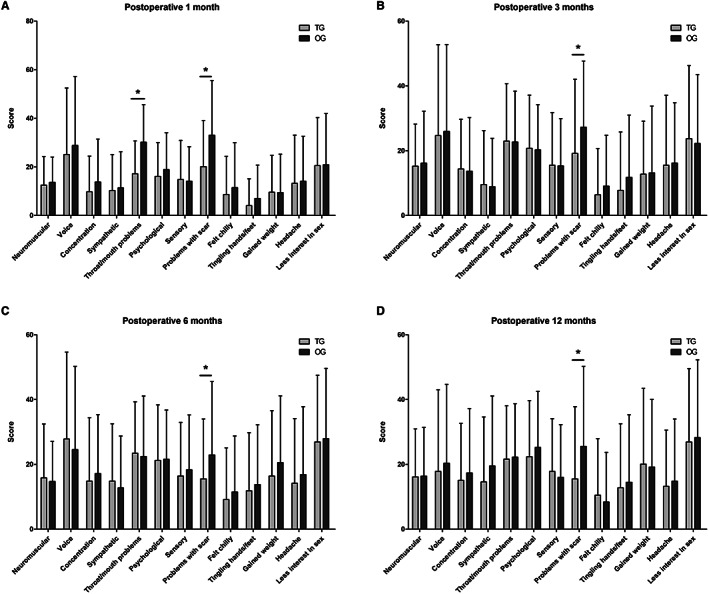
THYCA‐QoL score comparison between patients with PTMC in the transaxillary group and the open group: the patients in the transaxillary group showed significantly lower scores than the open group in two domains of HRQoL at 1 month postoperatively and in one domain at 3, 6, and 12 months postoperatively

The THYCA‐QoL questionnaire showed that some parameters in the transaxillary endoscopy group were better than those in the conventional open surgery group (Table 2). The “throat/mouth problems” scale score of patients in the transaxillary group was lower than that in the open group at 1 month after surgery, whereas no differences were observed between the two groups at 3, 6, and 12 months after surgery. In all follow‐up points, the scores of the transaxillary group in the “problems with scarring” item were lower than those in the open group, indicating that the patients in the transaxillary group had fewer complaints about scars (Table 2 and Figure 4).

**TABLE 2:** Comparison of quality of life in patients with papillary thyroid microcarcinoma in transaxillary group and open group by using THYCA‐QOL at 1, 3, 6, and 12 months postoperatively

THYCA‐QoL	Postoperative 1 month			Postoperative 3 months			Postoperative 6 months			Postoperative 12 months		
	TG	OG	*p*value	TG	OG	*p*value	TG	OG	*p*value	TG	OG	*p* value
Multi‐item scales												
Neuromuscular												
Cramp legs; felt slowed down; pain joints, muscles	12.48 ± 11.85	13.69 ± 10.39	0.477	15.22 ± 13.03	16.16 ± 16.05	0.682	15.83 ± 16.66	14.70 ± 12.38	0.611	16.13 ± 14.82	16.39 ± 15.00	0.913
Voice												
Hoarseness, weak voice	25.11 ± 27.38	28.79 ± 28.45	0.396	24.66 ± 28.07	25.93 ± 26.86	0.764	27.85 ± 26.80	24.58 ± 25.68	0.418	17.81 ± 25.20	20.37 ± 24.35	0.502
Concentration												
Attentional problems, difficulty thinking	9.82 ± 14.66	13.80 ± 17.66	0.118	14.38 ± 15.29	13.64 ± 16.56	0.763	14.84 ± 19.56	17.17 ± 18.20	0.422	15.07 ± 17.60	17.34 ± 19.91	0.439
Sympathetic												
Hot flushes, sensitive heat	10.27 ± 14.86	11.44 ± 14.80	0.609	9.59 ± 16.65	8.92 ± 14.93	0.783	14.84 ± 17.69	12.79 ± 15.94	0.428	14.61 ± 20.02	19.53 ± 21.57	0.130
Throat/mouth problems												
Problems swallowing, lump in throat, dry mouth	17.20 ± 13.49	30.19 ± 15.47	**0.000** ^*^	22.98 ± 17.70	22.67 ± 15.71	0.903	23.44 ± 15.88	22.44 ± 18.64	0.714	21.61 ± 16.45	22.22 ± 16.50	0.811
Psychological												
Anxious, restless, palpitations, abrupt tiredness	16.10 ± 13.98	18.85 ± 15.27	0.227	20.78 ± 16.38	20.29 ± 13.94	0.833	21.23 ± 17.13	21.55 ± 15.20	0.899	22.37 ± 17.34	25.25 ± 17.23	0.282
Sensory												
Eye problems, skin problems	14.84 ± 16.09	14.14 ± 14.15	0.763	15.53 ± 16.28	15.32 ± 14.61	0.931	16.43 ± 16.55	18.35 ± 16.91	0.461	17.81 ± 16.28	15.99 ± 16.31	0.471
Single‐item scales												
Problems with scar	20.09 ± 19.04	33.00 ± 22.59	**0.000** ^*^	19.18 ± 22.85	27.27 ± 20.41	**0.016** ^*^	15.53 ± 18.49	22.90 ± 22.66	**0.024** ^*^	15.53 ± 22.28	25.59 ± 24.66	**0.007** ^*^
Felt chilly	8.68 ± 15.74	11.45 ± 18.54	0.304	6.39 ± 14.33	9.09 ± 15.66	0.249	9.13 ± 15.97	11.48 ± 17.28	0.371	10.50 ± 17.46	8.42 ± 15.31	0.407
Tingling hands/feet	4.11 ± 11.03	7.07 ± 13.70	0.131	7.76 ± 18.02	11.78 ± 19.23	0.166	11.87 ± 17.89	13.80 ± 18.45	0.493	12.79 ± 19.74	14.48 ± 20.84	0.591
Gained weight	9.59 ± 15.19	9.43 ± 15.82	0.946	12.79 ± 16.32	13.13 ± 20.66	0.906	16.44 ± 20.13	20.54 ± 20.60	0.194	20.09 ± 23.40	19.19 ± 20.81	0.791
Headache	13.24 ± 19.83	14.14 ± 18.50	0.760	15.53 ± 21.58	16.17 ± 18.66	0.836	14.16 ± 19.97	16.84 ± 20.96	0.399	13.24 ± 17.34	14.81 ± 19.18	0.581
Less interest in sex	20.55 ± 19.74	20.88 ± 21.07	0.918	23.74 ± 22.55	22.22 ± 21.30	0.652	26.94 ± 20.53	27.95 ± 21.67	0.759	26.94 ± 22.67	28.28 ± 23.98	0.711

Abbreviations: OG, open group; TG, transaxillary group; THYCA‐QoL, thyroid cancer‐specific quality of life.

### 3.3 Statistical results of POSASquestionnaires

**FIGURE 5:**
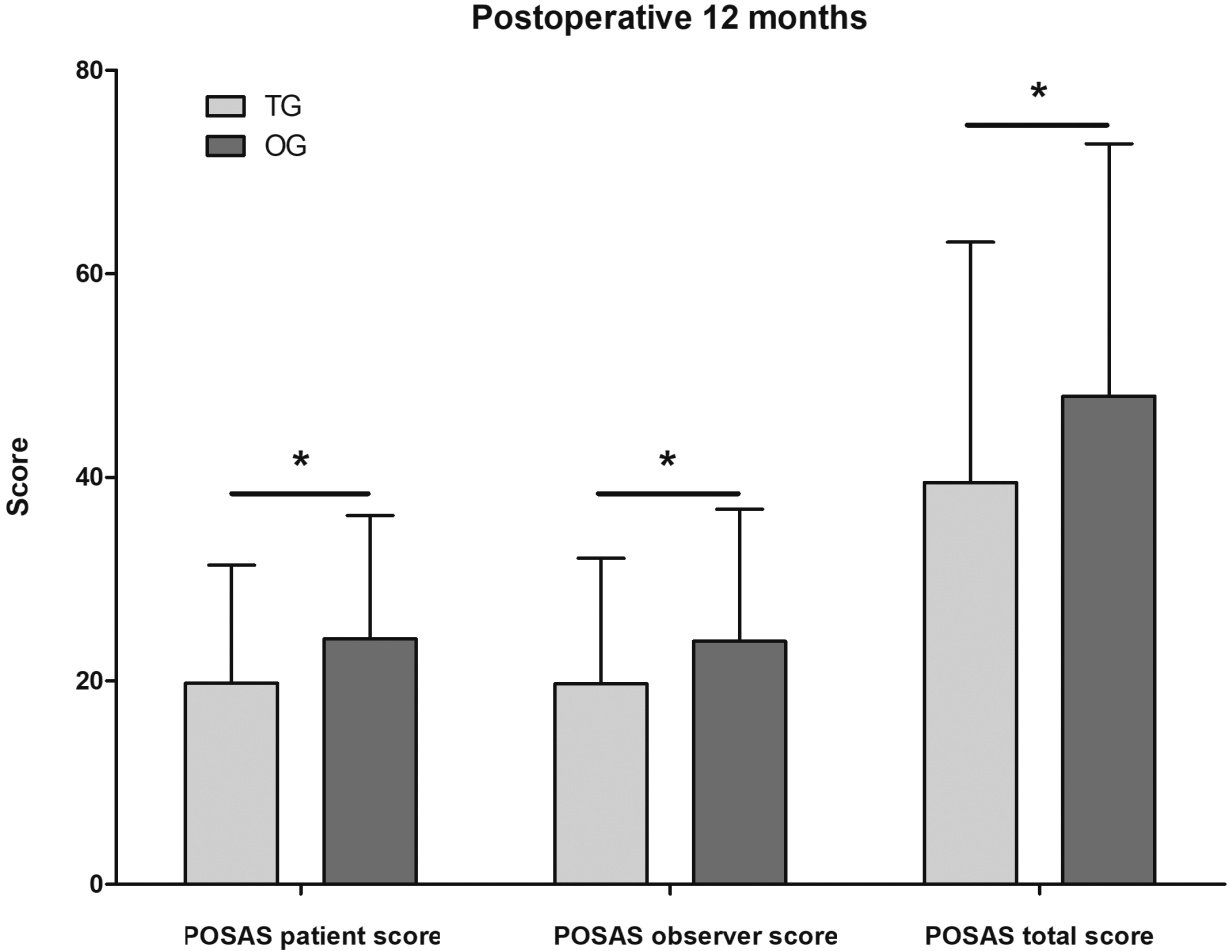
POSAS score comparison between patients with PTMC in the transaxillary group and the open group: the patients in the transaxillary group showed significantly lower scores than the open group in three domains at 12 months postoperatively

**FIGURE 6:**
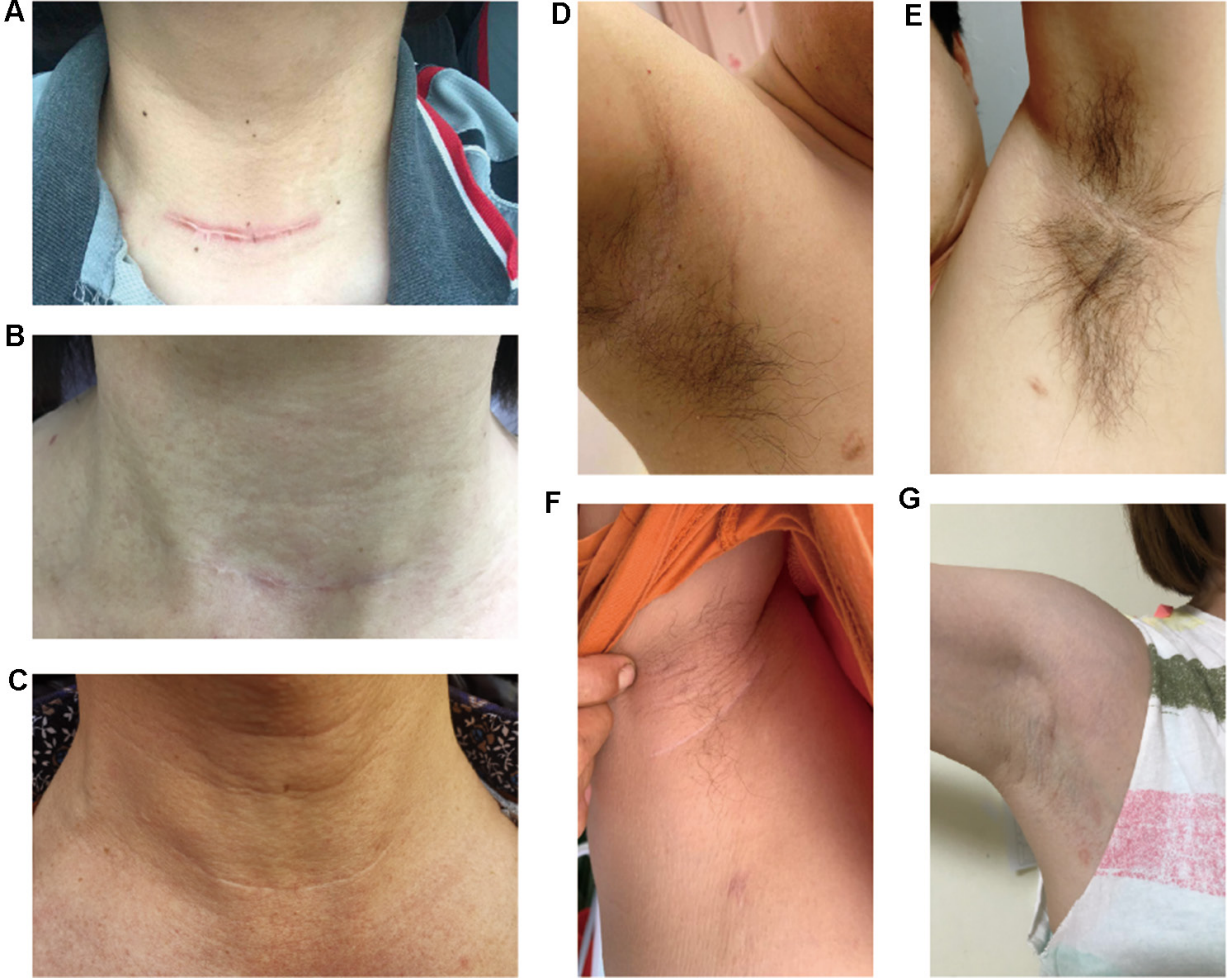
Representative scar pictures in two groups: A and B, open group; C, D, E, and F, transaxillary group

At 12 months after surgery, the transaxillary group scored better in all POSAS questionnaire items than the open group (Table 3). The open group had significantly higher POSAS patient score, POSAS observer score, POSAS total score, patient total score, and observer total score than the transaxillary group (Table 3 and Figure 5). The cosmetic effect of scars was significantly better in the transaxillary endoscopy group after 12 months (Figure 6).

**TABLE 3:** Comparison of cosmetic outcomes in patients with papillary thyroid microcarcinoma in transaxillary group and open group by using POSAS scores at 12 months postoperatively

Characteristics	Transaxillary group (*n* = 73)	Open group (*n* = 99)	*p* value
POSAS patient score	19.78 ± 11.58	24.10 ± 12.14	**0.02** ^*^
POSAS observer score	19.71 ± 12.34	23.89 ± 12.99	**0.035** ^*^
POSAS total score	39.49 ± 23.62	47.99 ± 24.79	**0.025** ^*^
Patient overall score	4.01 ± 2.48	4.88 ± 2.50	**0.026** ^*^
Observer overall score	3.84 ± 2.50	4.73 ± 2.68	**0.028** ^*^

Bold and * indicates the important values of *p*<0.05.

## 4 DISCUSSION

Our study introduced direct clinical results comparing transaxillary endoscopic lobectomy and conventional open lobectomy in terms of HRQoL and cosmetic effects in more patients and in longer postoperative follow‐up times. Even in the long term, transaxillary endoscopic lobectomy causes fewer postoperative scar‐related problems.

Transaxillary endoscopic lobectomy has been proven to be a safe and effective surgical method not only for benign thyroid disease but also for PTMC.^10^, ^15^, ^16^ Our study showed no statistical differences between the transaxillary and open groups in terms of blood loss volume, tumor size, positive central lymph nodes, and complications. According to previous studies, the incidence of RLN palsy after thyroid surgery is approximately 0%–18.6%.^24^, ^25^, ^26^ In our study, two patients in the transaxillary group and five patients in the open group had postoperative vocal cord dysfunction. All seven patients recovered within 4 weeks after surgery. We believe that the recurrent laryngeal nerve is dissected more clearly during transaxillary surgery, thus reducing the probability of nerve injury. As mentioned in previous studies, operating through the axilla can decrease the chance of touching the recurrent laryngeal nerve, thus preventing visible trauma to the recurrent laryngeal nerve.^12^, ^27^ However, as pointed out in previous studies, the average operative time and length of postoperative hospital stay were significantly longer in the transaxillary group due to the complexity of cavity building and the use of an endoscopic approach.^8^, ^28^ In addition, more patients in the open group experienced throat discomfort at 1 month postoperatively, which was gradually relieved and was considered to be related to the injury of the anterior cervical muscle during open surgery. Overall, we believe that endoscopic thyroid surgery via the transaxillary approach is a safe and minimally invasive technique for PTMC.

The recent American Thyroid Association Guidelines point out the importance of surgeons’ consideration of the long‐term QoL after surgery when making treatment decisions regarding the type of surgery.^4^ Health requirements change over time. It not only refers to the absence of disease but is also considered a state of complete physical, emotional, and social well‐being.^29^ The HRQoL has been widely used to assess personal health outcomes.^30^ Our study compared the HRQoL of patients with PTMC who underwent two types of surgery. Several THYCA‐QoL domains evaluated were significantly different between the transaxillary and open groups at four follow‐up time points, especially in terms of scars appearance. This result is consistent with that reported by Huang et al.(2016).^8^ At the same time, a large subcutaneous surgical wound caused by cavity creation did not reduce the QoL of patients in the transaxillary group. Therefore, endoscopic thyroid surgery via the transaxillary approach is feasible for PTMC patients to improve their QoL.

In our study, patients who underwent open surgery had more common scar problems than those who underwent transaxillary surgery. This is one of the main reasons for the decline in the QoL in the open surgery group. Previous studies have revealed that obvious scarring may negatively affect the QoL of patients with PTMC.^9^, ^31^ As most patients with thyroid cancer are women and the prognosis of the disease is good, a considerable number of female patients worry about the formation of a noticeable scar on the anterior neck. Although open thyroid surgery is safe and recommended, concerns regarding the appearance of scars may affect the QoL of patients after surgery. Problems with a noticeable scar include difficulty in choosing clothes, fear of communication with others, becoming inferior, and even affecting career development.^32^ The Chinese THYCA‐QoL questionnaire used in this study included questions related to scar problems. We found that the “problems with scarring” scale scores of the transaxillary group were lower than those of the open group in all four follow‐up points. To better assess the scarring in the two groups of patients, a further questionnaire survey was conducted using POSAS. After a 12‐month follow‐up evaluation, the POSAS scores of the two groups were comparable. The transaxillary group had more advantages than the open group in all aspects of POSAS. The transaxillary group did not develop hyperplastic scars, which was probably due to the low tension of the surgical incision.^33^ Because of its good concealment and lack of scar hyperplasia, the cosmetic effects of transaxillary surgery have been recognized by patients with PTMC. Our main result is similar to that of Lee et al.(2020), who showed that transaxillary thyroidectomy has a better cosmetic effect compared with that traditional open surgery.^34^ Consequently, our study confirms that transaxillary endoscopic lobectomy is superior to open surgery in terms of reducing scar problems, which could be the main reason for the improvement in HRQoL of PTMC patients in the transaxillary group.

### 4.1 Study limitations

Due to the limitations of our study, the results may be biased. First, the total number of thyroid cancer patients included in this study was limited. Second, the follow‐up period after surgery was only 1 year, which is relatively short. This may have led to an overestimation of the negative impact of surgery on the QoL. Over time, the QoL of patients with cancer may gradually improve.^22^ Finally, we did not perform a statistical analysis of the QoL of the two groups prior to surgery. Therefore, a prospective study with large sample size and long‐term follow‐up is warranted.

In addition, patients with uncontrolled chronic diseases and other cancer types were excluded, which limited the external validity of this study. Moreover, patients’ attitudes toward postoperative appearance may affect the choice of surgery and lead to some bias. All the above issues need to be investigated in‐depth and detail.

## 5 CONCLUSION

In conclusion, our study shows that transaxillary surgery has obvious advantages in terms of HRQoL and cosmetic effects, which is mainly due to the alleviation of scar problems. The role of transaxillary surgery as an alternative strategy for patients with PTMC is worthy of recognition.

## CONFLICT OF INTEREST

Drs Tingting Li, Zhicheng Zhang, Weisheng Chen, Shitong Yu, Baihui Sun, Xiangqian Deng, Junna Ge, Zhigang Wei, Shangtong Lei, and Guoxin Li have no conflict of interest or financial ties to disclose.

## AUTHORS’ CONTRIBUTION

Li TT and Zhang ZC contributed equally to the design and preparation of this study and should be considered co‐first authors; Li TT, Zhang ZC, Lei ST, and Li GX designed the research; Wei ZG, Ge JN, and Lei ST performed the surgeries and collected the data; Li TT, Chen WS, Yu ST, Sun BH, and Deng XQ analyzed the data; Li TT, Zhang ZC, and Li GX wrote the paper.

## Data Availability

The data that support the findings of this study are available on request from the corresponding author. The data are not publicly available due to privacy or ethical restrictions.
